# Recombinant Attenuated *Salmonella enterica* as a Delivery System of Heterologous Molecules in Cancer Therapy

**DOI:** 10.3390/cancers14174224

**Published:** 2022-08-30

**Authors:** Elayne Irene Becerra-Báez, Sergio Enrique Meza-Toledo, Paola Muñoz-López, Luis Fernando Flores-Martínez, Karla Fraga-Pérez, Kevin Jorge Magaño-Bocanegra, Uriel Juárez-Hernández, Armando Alfredo Mateos-Chávez, Rosendo Luria-Pérez

**Affiliations:** 1 Unit of Investigative Research on Hemato-Oncological Diseases, Children’s Hospital of Mexico Federico Gomez, Mexico City 06720, Mexico; 2 Departamento de Bioquímica, Escuela Nacional de Ciencias Biológicas, Instituto Politécnico Nacional, Mexico City 11340, Mexico; 3 Departamento de Microbiología, Escuela Nacional de Ciencias Biológicas, Instituto Politécnico Nacional, Mexico City 11340, Mexico; 4 Department of Molecular Biomedicine, Center for Research and Advanced Studies of the National Polytechnic Institute, Mexico City 07360, Mexico

**Keywords:** *Salmonella enterica*, cancer therapy, immunotherapy

## Abstract

**Simple Summary:**

Cancer is among the main causes of death of millions of individuals worldwide. Although survival has improved with conventional treatments, the appearance of resistant cancer cells leads to patient relapses. It is, therefore, necessary to find new antitumor therapies that can completely eradicate transformed cells. Bacteria-based tumor therapy represents a promising alternative treatment, particularly the use of live-attenuated *Salmonella enterica*, with its potential use as a delivery system of antitumor heterologous molecules such as tumor-associated antigens, cytotoxic molecules, immunomodulatory molecules, pro-apoptotic proteins, nucleic acids, and nanoparticles. In this review, we present the state of the art of current preclinical and clinical research on the use of *Salmonella enterica* as a potential therapeutic ally in the war against cancer.

**Abstract:**

Over a century ago, bacterial extracts were found to be useful in cancer therapy, but this treatment modality was obviated for decades. Currently, in spite of the development and advances in chemotherapies and radiotherapy, failure of these conventional treatments still represents a major issue in the complete eradication of tumor cells and has led to renewed approaches with bacteria-based tumor therapy as an alternative treatment. In this context, live-attenuated bacteria, particularly *Salmonella enterica*, have demonstrated tumor selectivity, intrinsic oncolytic activity, and the ability to induce innate or specific antitumor immune responses. Moreover, *Salmonella enterica* also has strong potential as a delivery system of tumor-associated antigens, cytotoxic molecules, immunomodulatory molecules, pro-apoptotic proteins, and nucleic acids into eukaryotic cells, in a process known as bactofection and antitumor nanoparticles. In this review, we present the state of the art of current preclinical and clinical research on the use of *Salmonella enterica* as a potential therapeutic ally in the war against cancer.

## 1. Introduction

In 1891, William B. Coley, a surgeon specializing in bone sarcomas, reported that treating his patients with malignancies with cultures of the bacterium responsible for erysipelas, eliminated the tumors [1]. These findings led to the development of “Coley’s Toxin”, a combination of the heat-inactivated bacteria *Streptococcus pyogenes* and *Serratia marcescens*, used until 1963; this extract was used in the treatment of sarcomas, carcinomas, lymphomas, melanomas, and myelomas. The development of radiotherapy and chemotherapy accounted for the gradual dismissal of Coley’s toxin [2]. Further studies (1976) using intravesical attenuated *Mycobacterium bovis*, the Bacille of Calmette-Guérin, proved to be the most effective in reducing the recurrence and progression of bladder cancer by triggering an inflammatory response. This approach remains in clinical use [3,4,5,6,7]. Although the use of bacteria in cancer treatment was abandoned for decades, recent advances in the fields of immunology and biotechnology have led to its resurgence [8]. Some of the advantages of bacterial cancer therapy in cancer management include: (a) its low cost, (b) its immunostimulatory activity (adjuvant); (c) its availability in biosafe strains, (d) a low incidence of side effects, and (e) its tendency to strictly colonize the tumor microenvironment [8]. To date, several bacterial genera have been reported to harbor antitumor activity and have been classified as: aerobic microorganisms such as the genus Mycobacterium; strict anaerobes such as Clostridium and Bifidobacterium; and facultative anaerobes such as those in the genera Escherichia, Listeria, Lactococcus, and Salmonella, among others. Salmonella, an intracellular facultative anaerobe bacillus, is the most fully studied bacterial genus in cancer treatment. In this genus, *Salmonella enterica* serovar Typhi (*Salmonella* Typhi) and *Salmonella enterica* serovar Typhimurium (*Salmonella* Typhimurium) can specifically target tumors and induce an antitumor response [7,8]. There is a great diversity of *Salmonella enterica-*attenuated strains that foster biosafety in preclinical assays and clinical trials [9].

## 2. Live-Attenuated *Salmonella enterica* Strains in Cancer Therapy

Various genes have been mutated to attenuate *Salmonella enterica* and minimize its virulence and pathogenicity, thus obtaining biosafe strains [10]. The reported mutations include: metabolic pathway auxotrophic mutations, virulence- and toxicity-associated mutations, and mutations that promote tumor selectivity and directionality toward the tumor. According to available information, auxotrophic mutations in metabolic pathways include those that compromise purine biosynthesis [11], and those affecting the biosynthesis of amino acids, including aromatic amino acids [12]; there are also combinations of several mutations [13] that preclude replication in unfavorable milieus lacking these molecular substrates, and hence, do not create greater risks in the host. These include Salmonella with mutations in the genes *aroA*, *aroC*, and *aroD* pertaining to the Shikimato pathway; these mutations prevent the production of aromatic amino acids, thus disabling the bacterium’s replication ability within the host, since these amino acids or their precursors are essential to its survival [6,14,15,16,17,18,19,20]. Other examples include bacteria that depend on the amino acids leucine and arginine [21,22,23,24,25,26,27]. Mutations have also been performed in *Salmonella enterica* genes that play a role in the biosynthesis of nucleic acids, such as the *purI* gene deletion [28,29] and mutations in the guaBA operon that negatively intervenes in the biosynthesis of guanine nucleotides [30,31]. 

Another group of mutations compromise the virulence-associated gene profile of *Salmonella enterica*, such as the mutation in the *msbB* gene [28,29] that interferes with the synthesis of lipid A, an indispensable component for lipopolysaccharide (LPS), the molecule associated with septic shock induced by *Salmonella enterica*; other mutations have been described in genes *faG*, *rdaF*, *dam* [32], *sptP* [33], and *gmd* [34] that also play a role in the formation of LPS [35], and mutations in genes *pagP8*, *pagL7*, and *lpxR9* that affect lipid A [36,37] in *Salmonella enterica*. Of special note are the mutations described in the *PhoP/PhoQ* genes [38], responsible for bacterial survival [39], and the mutations in the *relA* and *spoT* genes involved in bacterial virulence [40,41,42]. 

*Salmonella enterica* mutations also foster tumor selectivity and directionality, as in the case of mutations in operon *znuABC* that encodes the high-affinity zinc receptor and maintains tumor selectivity [43]. Other mutations are present in genes associated with adenylate cyclase, necessary for adaptation to osmotic gradients [44].

Research that has focused on the creation of safer strains while still maintaining tumoral tropism, has led to various gene mutations, such as mutant attenuations of genes *Pur*, *Ilv*, *Arg* or *Pur*, *Ilv*, *Ura* [45], or the *Salmonella enterica* recombinants mutated in genes *aroA* and *hisG*, in combination with deletions in the *cheY* gene associated with chemotaxis; the *fiGHI* gene associated with motility; the genes *invG*, *phoP*, *sseD*, and *ssrB* associated with invasiveness; and the gene *purA* associated with metabolism [46]. Finally, commercially available strains with vaccine purposes have also been used; they have been mutated in the *Gal E* gene and lack the Vi antigen [47]. In general, the described mutations have permitted the generation of biosafe attenuated strains with proven antitumor activity in preclinical and clinical trials. Table 1 summarizes some of the *Salmonella enterica*-attenuated strains used in antitumor therapy.

## 3. Selectivity and Permanence of *Salmonella enterica* in the Tumor Microenvironment

**Figure 1:**
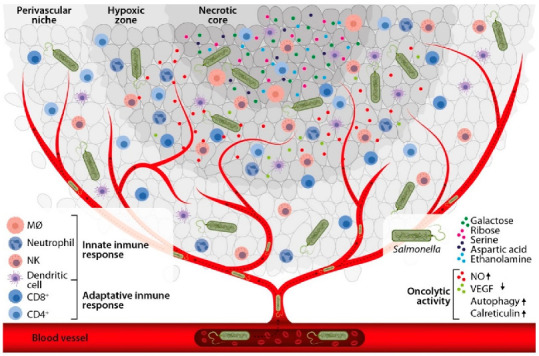
Tumor selectivity and inherent antitumor activity of *Salmonella enterica*. After *Salmonella enterica* enters the host through the mucosas or intravenously, it can specifically target tumor tissue due to hypoxia, acidity, necrosis, suppressed immune response, and the presence of chemoattractants of the bacterium in the tumor microenvironment. Subsequently, *Salmonella enterica*, activates inherent antitumor mechanisms, including its oncolytic activity per se, and the induction of the innate and adaptive immune responses.

Several studies have documented that *Salmonella enterica* has the ability to specifically target tumors [48,49], but how this tropism is not completely understood [45]. The tumor microenvironment is characteristically hypoxic (≤10 mmHg) [50], acidic [10], necrotic [10], and one in which the immune response is suppressed [5], but favoring the selectivity and permanence of *Salmonella enterica* within the tumor microenvironment [10]. Kasinskas et al., demonstrated that *Salmonella* Typhimurium accumulates in the quiescence zone, possibly sensing certain chemical secreted by tumor cells [51]. Subsequent studies have suggested that some molecules such as serine, aspartate, ribose and galactose possess chemotactic activity [51,52]. Recently, ethanolamine, a component used by Salmonella to colonize the gastrointestinal tract, has been found to be overexpressed in different types of neoplasias, and may also act as a chemoattractant of the bacterium towards the tumor [53,54,55]. After the bacteria has detected these chemotactic factors, systems involved in motility such as CheA/CheY are essential to colonization efficiency and bacterial distribution in malignant tissues [51,52,56,57,58]. However, other studies have suggested that motility-promoting bacterial proteins are not involved in *Salmonella enterica* tumor tissue colonization [46,55,59]. The accumulation of *Salmonella enterica* in tumor tissue has also been associated with impaired innate and adaptive antitumor immune responses [60,61,62]. In addition, *Salmonella* colonizing tumors, form biofilms as protection against phagocytosis by immune or tumor cells [63]. Other studies have stated that the aromatic amino acid (*aroA*) and purine (*purA*) pathways of *Salmonella enterica* are also of relevance in the bacterium’s establishment within the tumor, since mutations in these metabolic pathways lead to a decrease in bacteria in the tumor tissue [46,59]. These mechanisms may explain the preferential accumulation of *Salmonella enterica* in the tumor rather than in normal tissue, and thus potentiate their antitumor activity (Figure 1).

## 4. Intrinsic Antitumor Activity of *Salmonella enterica*

*Salmonella enterica* inherent or intrinsic antitumor activity has been demonstrated in different preclinical and clinical studies [4,5]. This activity has been evaluated in several cancer models, for instance: (a) murine cancer models, including sarcomas [4], leukemia [64], colon cancer [46,64], prostate cancer [65], T-cell metastatic lymphoma [66], B-cell lymphoma [67], breast and colorectal cancer [68], among other. In this models the administration of *Salmonella enterica* reduced the tumor growth; (b) murine models of xenotransplantation human cancer, including prostate and breast cancer [21,24,68,69], osteosarcoma [25], pancreatic cancer [26], and spinal gliomas [27], among others; most results report that *Salmonella enterica* inhibit tumor growth, increasing survival in these murine models, (c) Patient-Derived Orthotopic Xenograft (PDOX) murine models [70,71], in which *Salmonella enterica* A1-R colonized and decreased the size of the tumor in metastatic colon cancer [70], osteosarcomas [72,73,74,75,76,77], and melanoma models [78,79,80,81,82]. Tumor cells that are refractory to drug therapy [72,73,75,76,79,82], and kinase inhibitors [74,80] were also eliminated by *Salmonella enterica* A1-R in PDOX murine models.

The intrinsic antitumor activity of *Salmonella enterica* has also been evaluated in clinical trials of solid tumors, using intravenous doses of the VNP20009 strain (bearing mutation in the *msbB* and *pur I* genes). Results revealed no side effects due to the bacterial infection, but only modest colonization with no significant antineoplastic activity [29].

The documented antitumor activity of *Salmonella enterica* may be further enhanced by the route of administration; for instance, there is no significant difference in the colonization rate nor in the decrease in tumor volume if it is administered intravenously or via the peritoneum in comparison with oral administration. However, the oral route appears to be safer but the intravenous route promotes greater antitumor activity [46,83].

The described studies document the intrinsic antitumor activity of *Salmonella enterica*, but the mechanisms through which the bacterium acts remain to be further elucidated; some authors have suggested that they involve the inactivation of death signaling pathways in the tumor cell, and the induction of the antitumor innate and adaptive immune responses.

### 4.1. Salmonella enterica Activates Death Domain Pathways in Tumor Cells

Once *Salmonella enterica* is incorporated into the tumor microenvironment, it triggers a series of mechanisms with the aim to eliminate the transformed cell. These include: (1) competition with cancer cells for nutrients [10], (2) the release of antitumor bacterial toxins [84,85,86,87,88], (3) decreased angiogenesis [88], (4) activation of autophagy [89,90], (5) an increase in the amounts of calreticulin [43,91]; (6) *Salmonella* also has the ability to inhibit the expression of metalloproteinase-9 (MMP-9), an enzyme associated with the degradation of the extracellular matrix, angiogenesis, and tumor progression, mediated by its interference in the AKT/mTOR pathway [92]; (7) it induces sensitization to drug therapy by decreasing the levels of glycoprotein P (GP) [93,94,95], and (8) it induces the expression of gasdermin-D (GSDM-D), exposure to calreticulin (CRT), and the release of high-mobility group proteins (HMGB-1) into the extracellular medium, thus activating immunogenic cell death, particularly pyroptosis; this process is mediated by the excision of caspase-1 and the subsequent release of IL-β [96].

### 4.2. Salmonella enterica Activates the Antitumor Innate Immune Response

Among the antitumor activities induced by *Salmonella enterica*, the immune response triggered in the host is of vital importance. Intracellular bacteria such as *Salmonella enterica* can infect non-phagocytic cells through the type 3 secretion system (T3SS). This mechanism may promote the elimination of tumor cells infected by the bacteria by presenting bacterial antigens on their cell surface capable of being recognized by Salmonella-specific T lymphocytes [97,98].

Initial immunotherapeutic studies of *Salmonella enterica* were conducted by Kurashige S. et al., using minicells (vesicles with no genomic DNA) in murine models (sarcoma [99] and T-cell lymphoma [100]). The results showed that the administration of minicells activated the innate immune response and promoted the eradication of transformed cells.

Several studies have shown that the antitumor activity of *Salmonella enterica* results from the recruitment of immune response cells such as neutrophils (PMN), macrophages (MΦ), natural killer cells (NK), dendritic cells (DC), and CD4+ and CD8+ T cells [19]; it also results from increased expression of IL-1β, TNF-α [41], and other proinflammatory cytokines such as IFN-γ [39] produced by CD11c+ cells and CD68+ macrophages, both associated with tumor regression. In addition, bone marrow-derived macrophages were able to produce inflammasome-related proteins, including NLRP, IPAF, and caspase 1 [42].

Many bacterial components, including pathogen-associated molecular pattern molecules (PAMPs), as well as the CpG sequences, lipopolysaccharide (LPS), and flagellin, have proven to harbor antitumor activity, once they are recognized by pattern recognition receptors (PRR), such as Toll-like receptors (TLRs), that allow the activation of the signaling pathways involved in the innate and adaptive immune responses. In this context, when LPS binds to CD14 and TLR4, it induces the expression of tumor necrosis factor (TNF-α) [101,102,103]. The presence of this cytokine promotes tumor blood vessel hemorrhages, thus allowing the infiltration of mature immune cells of myeloid (PMN and MΦ cells) [104] and lymphoid (T, B and NK cells) [105,106,107] lineages, with the purpose of eliminating tumor cells. Another bacterial component recognized through the TLRs is flagellin, a sub-unit of the protein of the bacterial flagella, that can directly suppress tumor cell proliferation via TLR5, and induce an antitumor response mediated by perforin-dependent NK cells [107,108]. Once flagellin has bound to TLR5, it signals and activates the NF-κB signaling pathway [109]; this, in turn, has been shown to increase the levels of Fas or TNF-α-mediated apoptosis in Jurkat cells [110]. The peri-tumoral administration of flagellin significantly inhibits tumor growth by increasing the levels of IFN-γ and IL-4 [111]. Others studies have documented the major role played by flagellin in antitumor activity using murine models of melanoma metastases [112], lymphoma, [107], and colon cancer [113].

### 4.3. Salmonella enterica Activates the Antitumor Adaptive Immune Response

The changes promoted by *Salmonella enterica* in the tumor microenvironment induce the transition from an immunosuppressed to an immunogenic phenotype [114]. This process involves breaking immune tolerance by impairing the levels of T-regulatory lymphocytes (Treg) in the tumor microenvironment via LPS and the Braun lipoprotein (Lpp) of *Salmonella enterica* [115]; and by decreasing the levels of the enzyme indoleamine 2,3-dioxygenase-1 (IDO1) [90], an enzyme that acts on tryptophan metabolism and is associated with the development of immune tolerance in T lymphocytes [116,117], thus preventing the formation of kynurenine and promoting the proliferation of T lymphocytes capable of recognizing and eliminating the tumor. Consequently, tumor cells infected by *Salmonella enterica* can process and present bacterial antigens to T lymphocytes that in turn, induce the elimination of the transformed cell in different types of solid tumors [19,21,118,119]. In the tumor microenvironment, *Salmonella enterica* induces the activation of the cells involved in the adaptive immune response such as B and T lymphocytes [120], and dendritic cells (DC), thus allowing the cross-presentation of tumor antigens [103,121]. *Salmonella* infection induces the upregulation of connexin 43 (Cx43) [122,123], a protein involved in the formation of the gap union between tumor cells, and DCs that allow the transfer of tumor cell pre-processed peptides to the DC, hence inducing cross-presentation and subsequent cross-priming of the specific tumor antigen [122,124]. Studies in a murine B-cell lymphoma model have documented that the administration of *Salmonella enterica* elicited an adaptive immune local and systemic antitumor response via CD8+ and CD4+ intratumor T cells, pro-inflammatory cytokines (IFN-γ and IL-12), and antitumor specific antibodies [19].

## 5. *Salmonella enterica* as a Delivery System of Heterologous Antitumor Molecules

**Figure 2:**
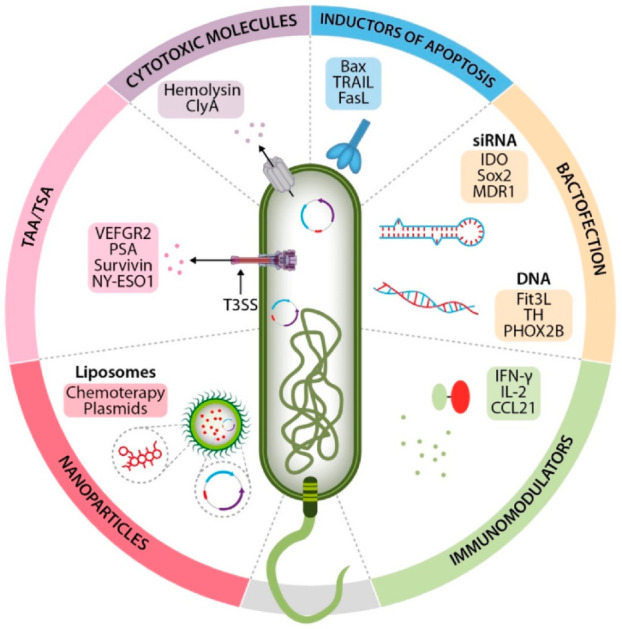
*Salmonella enterica* as a delivery system of heterologous molecules in cancer therapy. *Salmonella enterica*’s tropism for the tumor microenvironment and antigen-presenting cells (APC), as well as its ability to become a molecule factory within the tumor microenvironment, has allowed its use as a delivery system of heterologous molecules with antitumor properties such as: tumor-associated antigens or tumor-specific antigens, cytotoxic molecules, immunomodulating molecules, inducers of apoptosis, nucleic acids, and nanomolecules.

As described, the reported mechanisms mediating the intrinsic oncolytic activity of *Salmonella enterica* have been effective in preclinical models [4,5,7]. The tumor microenviroment (TME) is characterized by a state of hypoxia, necrosis, acidity, and suppression of the immune response; it facilitates migration and infection by *Salmonella enterica*, as well as its permanence in the tumor microenvironment for sufficient time (weeks) to activate its intrinsic oncolytic properties, revert immune tolerance, and induce tumor cell elimination. This is unlike the state of *Salmonella enterica* established in target organs such as the spleen or liver, where the bacterium begins to be eliminated within a week [125]. Also, to prevent the elimination of *Salmonella enterica* by the induced immune reaction and promote its establishment in the TME, consecutive and progressively greater CFU doses have been proven to be effective [126]. However, this intrinsic activity in initial clinical studies has not been sufficient to destroy the tumor [29,46,127,128] and has led to the development of strategies that potentiate *Salmonella enterica’*s oncolytic activity. Considering that once *Salmonella enterica* reaches the tumor, it becomes a complete factory producing antitumor heterologous molecules [10], its potential antitumor activity has been explored when used as a carrier of antitumor heterologous molecules such as: tumor-associated or tumor-specific antigens (TAA/TSA); cytotoxic molecules; immunomodulating molecules; inducers of apoptosis, nucleic acids, and nanomolecules (Figure 2). 

These loads were incorporated into the bacteria with recombinant DNA technology [129], and displayed in different *Salmonella enterica* compartments [120,121,122]. Table 2 outlines how live-attenuated *Salmonella enterica* strains act as a delivery system of biomolecules in cancer therapy.

### 5.1. Delivery of Tumor-Associated Antigens/Tumor-Specific Antigens

Tumors possess the ability to use proteins to promote cell transformation and tumorigenesis in order to establish a malignant phenotype; these molecules are known as Tumor-Associated Antigens (TAA) or Tumor-Specific Antigens (TSA), many of which are immunogenic and can be recognized by lymphocytes [185]. Considering *Salmonella enteric* a’s tropism for the tumor microenvironment and antigen-presenting cells (APC), various TAA/TSA have been coupled with *Salmonella enterica* proteins for transportation to the APC, and thus induce an antitumor immune response [7,10,186]. For instance, the use of *Salmonella* Typhimurium for delivery of Prostate Specific Antigen (PSA) via the Type 1 Secretion System (T1SS) has been reported, and it induced a decrease in tumor mass and the presence of antigen-specific CD8+ T lymphocytes [130]. Another antigen transported by *Salmonella enterica* and secreted by T1SS is the C-Raf antigen that not only plays a role in signal transduction but is also implicated in the developing malignant tumors. Immunization with this recombinant Salmonella in a murine pulmonary adenoma model, stimulated a CD8+ T cell response, induced specific anti-C-Raf antibodies, and decreased tumor mass [16]. Examples in which the T3SS has been used to transport TAA/TSA include studies in which Salmonella transports and secretes peptide 217–225 of Listeria monocytogenes protein 60 via the T3SS; its administration in a murine fibrosarcoma model induced an effector CD8+ T cell response, it decreased tumor size, and protected against challenge with an aggressively growing fibrosarcoma [33,132]. Immunization in an induced murine fibrosarcoma model with *Salmonella* Typhimurium with the ability to release the NY-ESO1 antigen (a protein in germ cells that is overexpressed in cancer of the lung, melanoma, esophagus, ovary, bladder and prostate) via T3SS, induced specific CD8+ and CD4+ T cell antigen-specific responses, and eliminated the tumor [133]. *Salmonella enterica* has also been used to translocate, via the T3SS, some antigenic determinants of proteins involved in angiogenesis [131] and oncogenic viruses [134]. Other secretion systems of gram negative bacteria such as the type 5 secretion system (T5SS) or autotransporters have also been used to express TAA/TSA, as in the case of a *Salmonella enterica* that expresses murine melanoma antigens through the AIDA-I autotransporter; the nasal administration of this recombinant bacterium stimulated an antigen-specific response of CD4+ and CD8+ T lymphocytes, increased lymphocyte proliferation, and induced the production of Th1 cytokines, as well as a decrease in tumor growth and metastases development [135]. Aside from the antigens expressed and secreted through the T1SS, T3SS, and T5SS systems in *Salmonella enterica*, other antigens have also been transported by this bacterium to APC or to the tumor microenvironment; these include survivin (a member of the inhibitor of apoptosis (IAP) protein family that promotes cellular proliferation and inhibits apoptosis), and proteins associated with tumor cell activation and migration [5,187]. The aforementioned studies document that the transport and release of TAA/TSA by *Salmonella enterica* represent a strategy to eliminate tumor cells and metastases.

### 5.2. Delivery of Cytotoxic Molecules

Various molecules with cytotoxic activity have been reported to harbor efficient antitumor activity, but a lack of selectivity of the tumor microenvironment that may lead to adverse effects remains an important challenge in cancer therapy. In this context, attenuated *Salmonella enterica* strains have been used to transport and release antitumor cytotoxic agents in situ, in an effectively and selectively manner [4,7,10]. *Salmonella enterica* can transport and release cytolysins originating in other bacterial species, as described in studies with *Salmonella* Typhimurium that express Hemolysin E (Hly E) and Cytolysin A (ClyA); in both cases, tumor invasion and tumor regression were documented in murine models of breast cancer and colon carcinoma, repectively. [138,139,140,153,188]. *Salmonella enterica* has also been used to transport enzymes that, once located in the tumor microenvironment, activate cytotoxic compounds (pro-drugs) that eliminate tumor cells [10]. For example, *Salmonella enterica* VNP20009 carrying the eukaryotic plasmid encoding purine nucleoside phosphorylase (sPNP) that convert the pro-drug 6-methylpurine-2′-desoxyriboside (6MePdR) into the toxic substance 6-methylpurine (6MeP), showed antitumor activity in tumor-bearing mice [141,142,189]. Other studies have described the antitumor efficacy of a recombinant *Salmonella* displaying both, a nanobody against CD20 antigen and the enzyme thymidine kinase, that activate an anticancer drug, using an in vivo model of non-Hodgkin lymphoma; treatment increased antitumor activity and improved survival in immunodeficient mice [143]. In this scenario and in view of the great number of molecules tested, it is imperative to improve the activity of pro-drug activating enzymes at appropriate sites. This is reflected when using the Y6 modified YieF enzyme obtained from *E. coli*, that significantly modified the activity of the pro-drugs Mitomycin C and 5-aziridinyl-2,4-dinitrobenzamide (CB 1954), requiring a lower enzyme concentration and hence, a decrease of HeLa cell viability in vitro [190]. Another pro-drug activating enzyme that has been produced and transported by Salmonella, is cytosine deaminase that can convert the pro-drug 5-fluorocytosine (5-FC) into 5-fluorouracil (5-FU), a cytotoxic metabolite used as treatment of gastric, breast, and head and neck cancers [191]. Cunningham et al., used *Salmonella enterica* VNP20009 TAPET-CD that expresses cytosine deaminase in clinical trials and reported promising results [192]. Other studies have documented the use of the *Salmonella enterica* VNP20009 strain to express and release the enzyme carboxypeptidase G2 (CPG2) that possesses a potentiated oncolytic effect in mouse melanoma, human breast, and colon carcinomas [144]. Another group of molecules that has also been expressed in *Salmonella enterica*, are immunotoxins. This combination of toxin and antibody are characterized by their binding to a receptor that is highly expressed in the tumor cell, allowing the toxin to enter the cell and cause defects in its physiology that lead to its death. Reports by Lim et al., reveal that a *Salmonella enterica* that expresses and releases the immunotoxic chimeric protein TGFα-PE38 [a fusion of transforming growth factor-alpha (TGF-α) and Pseudomona endotoxin A (PE38), decreased tumor size and increased survival in colon, cervix, and breast cancer murine models while also increasing these animals’ survival [145].

### 5.3. Delivery of Immunomodulating Molecules and Apoptosis Inducers

Although the use of live-attenuated bacterial vectors in the treatment of cancer represents a form of antitumor therapy per se, its effect has been potentiated by using *Salmonella enterica* as a transporter of immunomodulating molecules with antitumor activity [45,62]. Some examples are reported in several studies that have demonstrated the release of chemokines and cytokines such as CCL21 [62]; IL-2 [44,146]; IL-4 [147]; IL-18 [148]; IL-24 [149]; IFN-γ [151]; and LIGHT, also known as a lymphotoxin [152] analogue, into the tumor microenvironment. These approaches have demonstrated antitumor effects in the original neoplasia and in metastases, and the tumor cells were eliminated by the presence of DCs, macrophages, neutrophils, NK cells, and lymphocytes. Human clinical trials using treatments with interferons (IFN) have increased survival in patients with melanoma, but this modality is highly cytotoxic and leads to adverse effects. On the other hand, several studies have demonstrated that attenuated *Salmonella enterica* does not cause systemic toxicity [62] and represents an effective and safe strategy to carry immunomodulatory molecules such as IFN-γ and TNF-α into the tumor microenvironment [151,155].

Several studies have documented the use of tumor-targeting Salmonella to deliver agents that induce apoptosis through a death receptor pathway, such as Fas Ligand [150,158], TNF-α [5], and TRAIL (Tumor Necrosis Factor-Related Apoptosis-Inducing Ligand) [150] in the treatment of solid tumors. Results revealed a decrease in toxic side effects on normal tissue cells, while high intratumor concentrations of these apoptosis inducers promoted antitumor activity. Mansour et al., reported a *Salmonella enterica* expressing anti-tumor protein Lipidated azurin, Laz, that induces apoptosis through an interaction with the tumor suppressor protein p53; this recombinant Salmonella induced apoptosis in vitro models of glioblastoma and breast cancer [156]. Recently, Mateos Chávez et al., used a *Salmonella* Typhimurium SL3261 to express and release, via the MisL autotransporter (T5SS), a permeable peptide of the BH3 domain of the pro-apoptotic protein Bax; the administration of this recombinant to a non-Hodgkin lymphoma xenotransplant murine model (NHL) decreased the tumor mass, and increased the mice’s survival. In that study, the elimination of the transformed cell was mediated by an increase in apoptosis and the induction of the antitumor immune response [6]. In general, the described studies state that the expression and secretion of immunomodulatory molecules and inducers of apoptosis through *Salmonella enterica* is a promising approach in the battle against cancer.

### 5.4. Delivery of Nucleid Acids (Bactofection)

Another particularity of live-attenuated *Salmonella enterica* as antitumor therapy is its proven ability to transfer nucleic acids into eukaryotic cells, in a process known as Bactofection [193,194]. The principle by which this transfer occurs is not clear, but in the case of attenuated *Salmonella enterica*, it implies invasion of the host and the bacterium’s permanence in the phagocytic vesicle; it subsequently dies due to metabolic attenuation and liberates the plasmid. Then, the plasmid crosses the vesicular membrane through an unknown mechanism and reaches the cell’s nucleus [195]. This ability has been evaluated in different cancer murine models, including melanoma, bladder cancer, and lung adenocarcinoma [4]. The plasmids used in bactofection contain sequences encoding TAA/TSA, immunomodulatory molecules, and interference RNAs against some protein associated with tumor development and progression. An example of bactofection of genes encoding TAA/TSA was evaluated in recombinant Salmonella murine models that carry genes such as *HPV16 L1*, which encodes the human papillomavirus type 16 capsid protein, a gene encoding the MTDH/AEG1-1 protein, an oncogene associated with angiogenesis that is overexpressed in 40% of patients with breast cancer; a gene encoding the 4-1IBBL molecules, a member of the TNF family; and a CEACAM 6, a cell adhesion molecule; in all cases, they led to a decrease in tumor mass and increased survival [4]. Another TAA/TSA gene that has been transferred by bactofection is *Flt3L* (tyrosine kinase membrane receptor type III). The use of *Salmonella enterica* with the *Flt3L* gene in a melanoma murine model inhibited tumor growth and increased survival. Lode et al. reported a protective effect against neuroblastoma after the inoculation of a *Salmonella enterica* that releases a plasmid-encoding tyrosine hydrolase antigens [161]. Likewise, effective protection was observed after a lethal challenge by tumor cells, and the tumor mass decreased after administering the *Salmonella enterica*, conducting the bactofection of the plasmid expressing the Hsp70-tumor-associated antigen complex (Hsp70-TAA) in mice with melanoma [163]. Following the same strategy, Stegantseva et al., administered a *Salmonella enterica* with a plasmid containing the PHOX2B gene, an antigen associated with neuroblastoma, to C57Bl/6 mice with murine neuroblastoma. Their results revealed that bactofection mediated by *Salmonella enterica* led to an exacerbated cytotoxic response in conjunction with IFN-γ production [196]. Guan et al. also observed a decrease in tumor growth when using a *Salmonella* enterica to release a plasmid expressing the protein apoptin in mice with cancer of the larynx. The protein apoptin has the ability to induce selective apoptosis in malignant cells without affecting normal cells [160]. Likewise, Shao et al. demonstrated that using a *Salmonella enterica* that releases the *RBM5* gene, also known as RNA union motif-5, decreases Bcl-2 expression, while the expression of Bax; TNF-α; escinded caspase −3, −8, and −9; and PARP proteins increased, resulting in improved apoptosis in a murine lung adenocarcinoma model [164]. There are also studies in which the species *Salmonella choleraesuis* performs the bactofection of a plasmid encoding thrombospondin-1 (*TSP-1*) [197] or endostatin [198], that inhibited tumor growth and prolonged survival in murine melanoma and bladder tumor model. Examples of the bactofection of genes encoding immunomodulatory genes are epitomized by the bactofection of plasmids with genes encoding interleukin-4 or interleukin-18, that induce a systemic increase in IFN-γ with proven efficiency in delaying tumor growth and prolonging survival in a murine model of melanoma [4]. Another example is the bactofection of the gene encoding interleukin-24 in a gastric cancer model; it led to tumor regression mediated by proinflammatory cytokines, the activation of apoptosis, and angiogenesis disruption [149]. Also, the administration of a Salmonella that releases the plasmid encoding IL-15 decreased the number of metastases, and improved tumor remission in a neuroblastoma murine model. In this same context, the ability of *Salmonella enterica* was evaluated in terms of gene silencing by bactofection of Short hairpin RNA (shRNA) or small interfering RNAs (siRNA) sequences directed against different genes such as *indolamine 2*,*3-dioxygenase 1* [165], *Sox2* [166], *MDR1* [167,168], *surviving* [169], *Bcl-2* [170], and *Stat-3* [39,171]. The results obtained with this strategy were the induction of apoptosis mechanisms [165,169], the suppression of metastases, inhibition of tumor growth [166,169], and the suppression of proteins associated with the transport of therapeutic agents such as vincristine, paclitaxel, and adriamycin out of the tumor cell [167,168].

### 5.5. Delivery of Nanomolecules

In the last decade, the rapid advances in the development of new nanomaterials have permitted, among many things, to develop new diagnostic and therapeutic strategies in cancer treatment. Nanoparticles such as polymers, micelles, liposomes, and metal particles have been used as antitumor agent carriers (chemotherapies, antibodies, peptides, DNA, etc.) due to their easy preparation and biological safety threshold [199,200]. However, there are few nanomaterials capable of perfusing the tumor due to the high interstitial pressure and the density of tumor stroma. Likewise, deep nanoparticle penetration in the tumor is quite limited and precludes the distribution of drugs in the tumor’s hypoxic nucleus [201]. Recent studies have begun to explore the concept of using bacteria as nanoparticle carriers (nanobiohybrids), which could provide a new alternative in cancer treatment [202]. To date, many studies have proven that using these vectors in conjunction significantly improves the delivery of therapeutic agents in areas that are difficult to penetrate with conventional treatments, and their effect is reflected in improved antitumor response. Further, this combination can potentially decrease the limitations of each therapy administered on its own. A variety of strategies have also been used to combat tumors with these hybrid vectors (carriers of chemotherapeutic agents, photothermal therapy, nanocatalytic therapy, vaccination, and radiosensitization) [202]. In the last years, numerous strategies have been developed to load *Salmonella enterica* with nanoparticles in order to actively carry them to tumors and trigger an antitumor response. The bioconjugation of streptavidin-biotin is one of the most frequently used since the strong non-covalent link between these two molecules allows the adherence of streptavidin-covered nanoparticles to the biotinylated surface of Salmonella. With this strategy, Suh et al. loaded *Salmonella* Typhimurium VNP20009 with nanoparticles of poly (lactic-co-glycolic acid) (PLGA). These researchers demonstrated that the conjugation of nanoparticles with this strategy did not hinder the performance of the intratumor transport of Salmonella, and that this hybrid system (bacteria-nanoparticle) improved the distribution of nanoparticles in the tumor area 100-fold in an in-vivo 4T1 murine breast cancer model [173]. Another commonly used strategy to load *Salmonella enterica* with nanoparticles is incubation and electroporation. With this strategy, Zoaby et al. were able to load *Salmonella* Typhimurium LT2 with liposomes loaded, in turn, with doxorubicin. They also demonstrated that their system could cross various media, invade cancer cells, and release doxorubicin, thus eliminating tumor cells (chemotherapy effect) and through Salmonella per se (antibiotic effect) [172]. Further, aside from these two strategies to load Salmonella with nanoparticles, there are others based on electrostatic interactions, covalent links, and adherence mediated by folic acid. The antitumor reaction of nanoparticle-loaded Salmonella may have different approaches depending on the composition and nanoparticle load. Mainly, nanoparticles are usually loaded with antitumor drugs (doxorubicin, docetaxel, and paclitaxel) [174,203]. For instance, Ektate et al. loaded *Salmonella* Typhimurium YS1646 with low temperature-sensitive liposomes (LTSL) loaded with doxorubicin with a streptavidin-biotin system to strictly release doxorubicin in the tumor by heating it with high-intensity focused ultrasound (HIFU). This strategy significantly improved the polarization of macrophages with the M1 phenotype, and the therapeutic efficacy in an in vivo murine colon cancer model [174]. These hybrid vectors may also be used for vaccination purposes. To develop an efficient oral DNA vaccine against cancer, Hu et al. loaded the external membrane of Salmonella with cationic nanoparticles via electrostatic interactions, carrying plasmid DNA that encoded VEGFR2. This vaccination strategy led to a remarkable activation of T lymphocytes and cytokine release. It also successfully inhibited tumor growth by suppressing angiogenesis and fostering tumor necrosis in an in vivo murine melanoma model [204]. These nanobiohybrids can also be used as photothermal therapy against cancer. Chen et al. loaded Salmonella YB1 with nanophotosensitizers (nanoparticles loaded with indocyanine green) via amide bonds, in order to develop a photothermal therapeutic modality. This new focus proved to be quite efficient and precise in eradicating tumors in an in-vivo murine melanoma model [175]. Finally, metallic nanoparticles with no charge can also be used by Salmonella enterica to radiosensitize tumors. For example, Kefayat et al. covered the *Salmonella* Typhi Ty21a vaccine strain with gold nanoparticles covered with folic acid for anchoring. This model provided superior delivery of the gold nanoparticles compared to the control (gold nanoparticles covered with folic acid), and better focalization into the central zones versus the periphery in an in-vivo murine colon cancer model [176]. These findings establish that coupling *Salmonella enterica* with nanoparticles represents a novel alternative with great potential in the antitumor therapy realm.

## 6. Combination of *Salmonella enterica* and Conventional Antitumor Treatments

The combination of *Salmonella enterica* with conventional antitumor treatments is a strategy aimed to potentiate tumor elimination; also, failure of conventional treatments justifies the generation and evaluation of alternative therapies that could potentially sensitize tumor cells. Hence, the intrinsic oncolytic activity capacity of *Salmonella enterica* was studied when combined with conventional therapies: for example, *Salmonella* Typhimurium was administered directly into the tumor in combination with the treatment for non-Hodgkin lymphoma (NHL): CHOP (Cyclophosphamide, Doxorubicin, Vincristine, and Prednisone) in a NHL murine model, and it revealed that *Salmonella enterica* induced innate and adaptive immune responses, and increased pro-inflammatory chemokine and cytokine expression [178]. Similar results were also observed in a pancreatic cancer xenotransplant murine model after intra-peritoneal administration of *Salmonella enterica* in combination with drugs with different sensitivity profiles (5-fluorouracil, gentamicin, and cisplatin) [180]. The administration of cisplatin in combination with *Salmonella* Typhimurium A1-R was evaluated in a melanoma xenotransplant murine model. The results showed that, at low cisplatin doses, a significant suppression of melanoma growth ensued [82]. Igarashi et al. used *Salmonella* Typhimurium A1-R in combination with cisplatin and a recombinant methioninase (rMETase) in an osteosarcoma xenotransplant murine model, and observed a cellular decrease in the tumor associated with tumor necrosis [72]. Yang et al. detected that the intraperitoneal administration of *Salmonella choleraesuis* in a breast cancer and melanoma murine model decreased the expression of glycoprotein 1, which is known to be a resistance protein to multiple drugs (MDR1). The same study reported that treatment with 5-fluorouracil significantly increased cell death in cells infected with *Salmonella enterica*, and increased apoptosis biomarkers [205]. Hiroshima et al. reported that the combination of *Salmonella* Typhimurium and trastuzumab, a humanized monoclonal antibody, in a cervical cancer murine model, led to a decrease in tumor mass, because approximately 70% of cancer cells were destroyed in the group of mice treated with *Salmonella* Typhimurium and trastuzumab [179]. Other studies have proven that the intravenous administration of *Salmonella enterica* combined with the transfer of T cells acts synergistically to eradicate the tumor in a fibrosarcoma murine model [183]. Aside from the aforementioned strategies, the use of *Salmonella enterica* in conjunction with other molecules such as caffeine (CAF) and valproic acid (VPA) has been studied in a pleomorphic rhabdomyosarcoma xenotransplant murine model. The results of the intravenous administration of Salmonella and CAF + VAP showed a decrease in cellularity, whereby the greatest effect was observed in the group treated with Salmonella in combination with CAF and VAP [184]. The recombinant strains of *Salmonella enterica* that express or carry antitumor heterologous molecules have also been evaluated in combination with other treatments. That is the case of *Salmonella enterica*, which releases siRNAs against the hypoxia-inducible factor α (HIF-1α), with the aim of improving the effect of cisplatin in a prostate cancer murine model. The results showed that eliminating HIF-1α improved the response of tumor cells to cisplatin since aerobic glycolysis was redirected to mitochondrial oxidative phosphorylation due to the overexpression of reactive oxygen species (ROS) [181]. Zhang et al. reported that the intraperitoneal administration of a *Salmonella enterica* that releases siRNA directed against ATP-binding cassette sub-family B member 5 (ABCB5), which confers drug resistance, in combination with paclitaxel or doxorubicin, decreased the expression of ABCB5 in the tumor, controlled tumor growth, and increased the mice’s survival [182]. Our group reported that in an in-vitro model of prostate cancer cells, bactofection induced by *Salmonella* enterica of a plasmid encoding a peptide in the BH3 region of the pro-apoptotic protein Bax, sensitizes prostate cancer cells to treatment with cisplatin [206]. Finally, Liu et al. reported that the intravenous administration of Salmonella Typhimurium, which produces cytotoxic protein A (ClyA) in the presence of radiotherapy, suppressed the tumor in a colon cancer murine model [177]. As documented, the combination of *Salmonella enterica* with conventional treatments leads to a synergistic antitumor effect that in most cases is associated with a decrease in the necessary concentration of the administered conventional therapy and suggests the possibility of hindering the secondary effects resulting from conventional treatments.

## 7. Clinical Trials Using *Salmonella enterica* as Cancer Treatment

Although preclinical studies have yielded promising results, cancer treatments mediated by *Salmonella enterica* have only been implemented in a small number of human clinical trials. However, based on preclinical successes, *Salmonella* Typhimurium VNP20009 with mutations in the *msbB* genes (affecting the formation of lipid A, and decreasing the toxicity associated to the lipopolysaccharide), and *purI* genes (turning it dependent on an external adenine source), entered phase I human clinical trials in 1999, and were administered intravenously to 24 patients with metastatic melanoma and 1 patient with metastatic renal cell carcinoma. The results showed that VNP20009 could be safely used in cancer patients and established that the maximally tolerated dose was 3 × 10^8^ CFU/m^2^. There was limiting dose toxicity in patients who received 1 × 10^9^ CFU/m^2^, including thrombocytopenia, anemia, persistent bacteremia, hyperbilirubinemia, diarrhea, vomiting, and nausea, among others. VNP20009 induced a dose-related increase in the concentration of circulatory pro-inflammatory cytokines, such as interleukin IL -1β, tumor necrosis factor alfa, IL-6, and IL-12. There were no antitumor effects and further studies were suggested to decrease this dose-related toxicity [29]. Subsequent studies modified the VNP20009 strain by inserting a gene encoding cytosine deaminase (CD) from *E. coli* to generate a strain named TAPET-CD [207]; this new strain was used in a pilot test in three patients with refractory cancer to investigate the viability and effectiveness of an intratumor injection of TAPET-CD [208]. However, results were not significant and adverse effects continued. Two patients had bacterial colonization in the tumor tissue that persisted for at least 15 days after the initial injection. In these patients, the conversion of 5-FC to 5-FU was demonstrated with a 5-FU tumor: plasma ratio of 3:1, with substantially higher 5-FU levels in the site colonized by the TAPET-CD strain [192]. Since most tumors are methionine-dependent, the VNP20009 strain has also been used to express L-methioninase (SGN1). SGN1 was designed as a tumor therapeutic bacterium that can replicate and preferentially accumulate in tumors and deprive them of essential amino acids after the administration of the oncolytic enzyme L-methioninase. A phase 1 study will be conducted in patients with advanced and/or metastatic solid tumors that have been histologically confirmed, refractory to standard curative therapy, and for which there is no other conventional therapy available [209,210]. The *Salmonella* Typhi Ty21a strain has also been used to carry, via a plasmid, the sequence encoding the vascular endothelial growth factor receptor 2 (VEGFR-2) and induce an immune response against this antigen. The modified Ty21a strain was used to immunize 45 patients with stage IV pancreatic cancer in an attempt to decrease the tumors’ neovascularization [126]. The vaccine named VXM01 was administered at a dose of 10^6^ to 10^10^ CFU and in the first stage of the study, the researchers observed that its administration is safe in pancreatic cancer patients. Later, in a second study with 26 patients, 16 were immunized with VXM01 and 8 with placebo; those that were immunized with VXM01 had a decrease in peripheral lymphocytes, an increase in neutrophils, and developed adverse effects of moderate to medium severity. However, at least 8 of the patients immunized with VXM01 developed specific T lymphocytes against VEGFR-2, which could lead to the patients increased lifespan [211]. These early clinical trials revealed data on the safety of administering *Salmonella enterica* to cancer patients. Their results indicated that a dose of 10^9^ Salmonella CFU can be excreted. In cases in which a patient is positive for bacterial excretion, antibiotic decontamination of the gastrointestinal tract is indicated; the patient’s feces must also be collected and incinerated to protect the environment and the study personnel [126,211]. Another phase II clinical trial, currently in the recruitment stage, will use a *Salmonella* Typhimurium χ4550 strain that contains the gene for human IL-2, a potent anticancer immune stimulant. This *Salmonella enterica*, named Saltikva, will be administered orally in addition to the standard treatment protocol, in patients above the age of 18 with metastatic stage IV pancreatic cancer [212]. In the previous phase I study, the researchers observed that the administration of this strain in a single escalating dose to patients with solid liver metastatic tumors did not cause adverse or toxic effects at a dose of 10^10^ CFU in the 22 treated patients; although survival did not increase, an increased circulating NK cell response was observed, suggesting an immune effect of treatment with Saltikva [213]. Table 3 summarizes the clinical trials using *Salmonella enterica* as cancer therapy.

## 8. Limitations

We have broadly documented the antitumor activity of *Salmonella enterica* in pre-clinical and clinical models; however, this strategy has inherent limitations such as the following:

(a) Biosafety. The attenuation of *Salmonella enterica* virulence factors was widely used to counteract its infectivity; but attenuation per se has been occasionally associated with a decrease in antitumor therapeutic efficacy [35,221]. It is imperative to establish a balance between decreased virulence and clinical efficacy. Several bacterial strains that initially yielded discouraging results have been improved in recent years, and have been shown to be much safer, as they maintain their tumor specificity, their antitumor efficacy has increased, and their toxicity in normal tissue has been minimized; this has optimized our ability to deliver antitumor therapeutic agents such as cytokines, cytotoxic drugs, tumor-associated antigens, and pro-drug enzymes [222].
(b) Routes of administration. The route of administration of *Salmonella enterica* is pivotal to this vector’s safety and antitumor activity since the systemic administration of bacteria may be highly toxic and lead to serious adverse effects. Oral administration is considered the safest route, but at the expense of increased toxicity, possible adverse effects resulting from infection, and jeopardizing therapeutic efficacy.
(c) Dose optimization. Since live bacteria proliferate in target tissues, an effective dose does not necessarily reflect the administered dose. An effective dose hinges on many factors such as the route of administration, the target tissues’ accessibility, the degree of vascularization, tumor immunogenicity, and the presence of infiltrating inflammatory cells in the tumor [222]. The administration of progressively escalating CFU doses appears to promote antitumor efficacy [126].
(d) Genetic instability. Genetically modified live bacteria that carry antibiotic-resistance genes or mobile genetic elements are entirely inadequate for clinical use since these recombinant elements may be transferred horizontally from the plasmids in the treatment bacteria, thus carrying the antibiotic-resistant genes to other genes in the host or environment. Further, the plasmids may become lost or mutate upon reaching the tumor tissue and trigger an exaggerated infectious response or treatment failure. This drawback may be overcome by integrating a gene expression cassette with no antibiotic-resistance genes into the bacterial chromosome to guarantee genetic stability [223,224].
(e) Control of bacterial growth in vivo. The uncontrolled growth and propagation of bacteria in the patient is of major concern, so alternatives that have been suggested include the incorporation of additional features to the modified strains, such as genetic “switches” that can guarantee bacterial containment [225,226,227]. Another approach consists in building lethal systems within the recombinant bacteria, the so-called “suicide genes”, that may specifically destroy the host bacterium without interfering with normal flora or therapeutic efficacy. Some attenuated *Salmonella enterica* strains are guaranteed to limit bacteria-derived infections, and should this fail, antibiotics can be used to eliminate persistent bacteria [41,228].
(f) Patient selection. In clinical trials, patients that do not respond to conventional treatment or that are refractory to current standard therapies tend to be the subjects of interest for *Salmonella enterica* treatment; but patient selection for the administration of this treatment must be very thorough. Immunocompromised patients with underlying conditions or therapies must be excluded to prevent uncontrollable and overwhelming bacterial infection and its migration beyond the tumor site. Some patients appear to be predisposed to infections beyond the treatment’s aim. For example, certain bacteria preferentially proliferate in necrotic tissue, as is the case after radiation or due to associated comorbidities. The administered bacteria could also potentially colonize injuries or implanted medical devices such as artificial joints or valves, among others [229]. This could be prevented by correctly determining the administered dose, the route of administration, the administration intervals, and the timely elimination of bacteria post-administration. Thus, the clinician must scrupulously evaluate potential treatment candidates.
(g) Pre-exposure and antibacterial immunity. One of the inconveniences of using bacteria as antitumor agents is the host’s immune response triggered when bacterial concentrations increase, and in the best case scenario, leads to the elimination of the introduced bacteria [229], leading to treatment failure. A possible solution would be the generation of optimized Salmonella strains with a greater immunostimulatory capacity and capable of overcoming the immunity resulting from bacterial pre-exposure [230]. Other proposed strategies suggest the encapsulation of *Salmonella enterica* with compounds that can prevent the binding of specific antibodies to the bacterium, and that do not hinder the bacteria’s ability to focus on the tumor [231]. The administration of escalating CFU doses appears to counteract antibacterial pre-immunity, thus permitting antitumor activity [126].
(h) Production of biological agents. The manufacturing of live bacteria is significantly more complex than that of small molecule antitumor drugs. Unlike small molecules or other non-viable clinical agents, live therapeutic bacteria cannot be sterilized by filtration or heating, and that is the main challenge when producing biologicals following good manufacturing practices. Currently, the manufacture of bacteria-based cellular therapies, as in the case of *Salmonella enterica*, is a regulated process centered on product safety, consistency, and stability. The FDA recently published detailed industry guidelines on the information that should be provided when developing bacteria- or virus-based biological products. Included is a list of every component used in the manufacturing process; the generation of a seed stock; the expansion and characterization of the microbial mother/stem cell bank; the absence of any lysogenic prophage; information on the genome sequence; and all, if any, chromosomal modifications, phenotypic confirmation of attenuation, microbial purity (clonality), cell viability and stability, among others [224,232].

## 9. Conclusions

A century after the systematic use of bacteria in antitumor therapy was documented, this modality underwent a resurgence after several decades of neglect. This therapeutic alternative has been renewed as a result of advances in tumor immunology and genetic engineering. In this context, live-attenuated *Salmonella enterica*, a facultative anaerobic bacterium, has been more broadly studied in antitumor therapy due to its proven tropism for the tumor microenvironment and antigen-presenting cells; the tumor microenvironment, characterized by hypoxia, necrosis, acidity, and suppression of the immune response, facilitates migration and infection by *Salmonella enterica*, and its permanence in the tumor microenvironment, where it activates its intrinsic oncolytic properties and induces an immune response that eliminates the tumor cell. However, although the described clinical trials in this review reveal efficient antitumor activity, the initial clinical trials have documented modest antitumor responses, but the use of *Salmonella enterica* as a delivery system of heterologous molecules that maximize antitumor activity is promising. Once *Salmonella enterica* reaches the tumor microenvironment, it has been described as a veritable factory of antitumor heterologous molecules. In the review, we detailed the usefulness of *Salmonella enterica* as a delivery system of tumor-associated antigens or tumor-specific antigens (TAA/TSA) of cytotoxic molecules, immunomodulating molecules, apoptosis-inducer molecules, nucleic acids, and nanomolecules, with very promising preclinical outcomes. We also described the ability of *Salmonella enterica* to sensitize tumor cells to chemotherapy, radiotherapy, and immunotherapy. The presented phase I or II clinical trials document the use of biosafe strains such as *Salmonella* Typhimurium strain VNP20009 and strain χ4550, as well as *Salmonella* Typhi strain Ty21a, that have proven to be safe, cause few adverse events in humans, and activate antitumor immune responses; however, their minor effects on the tumor mass and on survival require further research in the clinical trials in course.

Finally, based on the documented preclinical and clinical studies in this review, we may conclude that live-attenuated *Salmonella enterica* is an excellent delivery system of heterologous molecules with antitumor properties and represents a promising therapeutic alternative in the fight against cancer.

## Figures and Tables

**Table 1:** Attenuated *Salmonella enterica* strains used in murine malignancy models.

Species	Strain	Mutation	Treated Malignancy	Reference
**Mutations in genes of metabolic pathways**				
*Salmonella* Typhimurium	SL3235	*aroA*	Plasmacytoma, Non-Hodgkin lymphoma	[6,11,12,13,14]
*Salmonella* Typhimurium	SL7207	*hisG46*, *DEL407 aroA544::Tn10 (Tc^s^)*	Lung cancer	[15,16]
*Salmonella* Typhimurium	YB1	*asd*	Hepatocellular carcinoma	[17]
*Salmonella* Typhimurium	LVR01	*aroC*	B cell lymphoma	[18,19]
*Salmonella* Typhimurium	BRD509	*aroA* and *aroD*	Murine melanoma	[20]
*Salmonella* Typhimurium	A1-R	*Leu*,*Arg*	Prostate cancer, Spinal glioma, Pancreatic cancer, and Fibrosarcoma	[21,22,23,24,25,26,27]
*Salmonella* Typhimurium	VNP20009	*purI*,*msbB*	Metastatic melanoma	[28,29]
*Salmonella* Typhi	CVD915	*guaBA*	Breast adenocarcinoma, T cell lymphoma	[30,31]
**Mutations in genes associated with virulence**				
*Salmonella* Typhimurium	VNP20009	*purI*,*msbB*	Metastatic melanoma	[28,29]
*Salmonella* Typhimurium	RE88	*aroA* and *dam*	Breast Carcinoma	[32]
*Salmonella* Typhimurium	SB824	*sptP::Kan*	Fibrosarcoma	[33]
*Salmonella* Typhimurium	ST8	*aroA::Tn10*, *gmd::Plac-T7RNAP*,*htrA::PpepT-asd-PsodA*, *infA::Ptet-tetR.*	Colon cancer	[34]
*Salmonella* Typhimurium	14028	*rfaL*, *rfaG*, *rfaH*, *rfaD*, *rfaP* and *msbB*	Colon cancer	[35]
*Salmonella* Typhimurium	S634	*pagP*, *pagL* and *lpxR*	Colon carcinoma	[36,37]
*Salmonella* Typhimurium	LH340	*PhoP/PhoQ*	Prostate cancer	[39]
*Salmonella* Typhimurium	ppGpp	*relA::cat*,*spoT::kan*	Colon adenocarcinoma	[40,41,42]
**Gene mutations associated with tumor selectivity**				
*Salmonella* Typhimurium	SA186	*znuABC*	Breast Adenocarcinoma	[43]
*Salmonella* Typhimurium	X^4550^	*Cya-1*, *Crp-1*	Adenocarcinoma	[44]
**Mutations in multiple genes**				
*Salmonella* Typhimurium	YS7211	*Pur*, *Ilv*, *Arg* and *Pur*, *Ilv*, *Ura*.	Melanoma	[45]
*Salmonella* Typhimurium	SL1344	*cheY*, *fliGHI*, *invG*, *phoP*, *sseD*, *ssrB*, *aroA*, and *purA*	Colon Carcinoma	[46]
*Salmonella* Typhi	TY21A	Chemical attenuation, UDP-glucose-4-epimerase	Murine bladder cancer	[47]

**Table 2:** *Salmonella enterica* as a carrier of antitumor heterologous molecules.

Species	Mutation	Heterologous Molecule	Type of Tumor in Murine Model	Generated AntiTumor Response	References
**Tumor-associated antigens/Tumor-specific antigens**					
*Salmonella* Typhimurium	*aroA*	PSA	Prostate cancer	Cytotoxic CD8+ T cells	[130]
*Salmonella* Typhimurium	*aroA*	VEGFR-2	Melanoma	Cytotoxic CD8+ T cells	[131]
*Salmonella* Typhimurium	*aroA*	C-RaF	Lung adenocarcinoma	Cytotoxic CD8+ T cells	[16]
*Salmonella* Typhimurium	*aroA*	Peptide 217-225 of protein P60	Fibrosarcoma	Effector CD8+ T cells	[33,132]
*Salmonella* Typhimurium	*phoP*, *phoQ*	NY-ESO1	Fibrosarcoma	Specific CD4+ and CD8+ T cells	[133]
*Salmonella* Typhimurium	*aroA*	E7 (HPV16E7)	Cervical cancer	INFγ and TNFα	[134]
*Salmonella* Typhimurium	*aroA*	Melan-A	Melanoma	Th1 and CTL response	[135,136]
*Salmonella* Typhimurium	*purD*, *htrA*	SVN	Colon cancer and lymphoma	Induction of CD8+ Treg cells	[137]
**Cytotoxic molecules**					
*Salmonella* Typhimurium	*waaN*, *purl*, *aroA*	HlyE	Breast cancer	Increased LDH	[138]
*Salmonella* Typhimurium	*ppGpp*	ClyA	Colon cancer and hepatocellular carcinoma	Decrease in tumor size	[139,140]
*Salmonella* Typhimurium	*aroA*, *purl*	PNP	Breast cancer	Increase in apoptosis	[141]
*Salmonella* Typhimurium	*purI*, *msbB*	PNP	Melanoma	Infiltration by CD8+ cells	[142]
*Salmonella* Typhimurium	*aroA*	HSV-TK	Lymphoma	Sensitivity of tumor cells	[143]
*Salmonella* Typhimurium	*pul*, *msbB* and *asd*	CPG2	Breast and colon cancer, melanoma	Cytotoxicity of tumor cells and inhibition of tumor growth	[144]
*Salmonella* Typhimurium	*ppGpp*	TGFα-PE38	Colon and breast cancer	Delay in tumor growth	[145]
**Immunomodulating molecules and apoptosis inducers**					
*Salmonella* Typhimurium	*Pur*, *msb*	CCL21	Breast Carcinoma	Inhibition of tumor growth	[62]
*Salmonella* Typhimurium	*Cya-1*, *Crp-1*	IL-2	Adenocarcinoma	Decreases metastases	[44,146]
*Salmonella* Typhimurium	*aroA*	IL-4, IL-18	Melanoma	Increases IFN-γ levels	[147]
*Salmonella* Typhimurium	*Pur*, *msb*	IL-18	Colon Carcinoma	Inhibits tumor growth	[148]
*Salmonella* Typhimurium	*aroA*	IL-24, Apoptina	Gastric cancer	Inhibits tumor growth	[149]
*Salmonella* Typhimurium	*aroA*	TRAIL, VP3	Gastric cancer	Increases caspase-3 and 9 expression	[150]
*Salmonella* Typhimurium	*aroA*, *aroD*	IFN-γ	Melanoma	Inhibition of tumor growth	[151]
*Salmonella* Typhimurium	*purI*,*msbB*	LIGHT	Breast Carcinoma	Inhibition of tumor growth	[152]
*Salmonella* Typhimurium	*relA::cat*, *spoT::kan*	TGFα-PE38	Breast and colon cancer	Inhibition of tumor growth	[145]
*Salmonella* Typhimurium	*relA::cat*,*spoT::kan*	L-asparaginasa	Colon Adenocarcinoma, pancreas, and breast cancer	Inhibition of tumor growth	[153]
*Salmonella* Typhimurium	*Cya-1*, *Crp-1*	IL-2	Osteosarcoma	Decrease in metastases	[154]
*Salmonella* Typhimurium	*aroA*, *aroD*	TNF-α	Melanoma	Induction of de apoptosis	[155]
*Salmonella* Typhimurium	*msbB*, *purI*	Laz	Glioblastoma	Induction of apoptosis	[156]
*Salmonella* Typhimurium	*purI*, *msbB*	FasL	Breast Carcinoma	Inhibition of tumor growth	[157]
*Salmonella* Typhimurium	*msbB*, *purI*	FADD	Melanoma	Induction of apoptosis	[158]
**Nucleid acids (Bactofection)**					
*Salmonella* Typhimurium	*aroA*, *aroD*	Flt3	Melanoma	Inhibition of tumor growth	[159]
*Salmonella* Typhimurium	*PhoP/PhoQ*	Apoptina	Larynx Cancer	Decreased cytotoxicity and increased apoptosis	[160]
*Salmonella* Typhimurium	*hisG46*, *DEL407* *aroA544::Tn10 (Tc^s^)*	Tirosina hidrolasa	Neuroblastoma	Protection against tumor challenges	[161]
*Salmonella* Typhimurium	*hisG46*, *DEL407 aroA544::Tn10 (Tc^s^)*	IL-15	Neuroblastoma	Tumor remission	[162]
*Salmonella* Typhimurium	*aroA*	Hsp70-TAA	Melanoma	Activation of T cells, tumor elimination	[163]
*Salmonella* Typhimurium	Chemical attenuation, UDP-glucose-4 epimerase	RBM5	Lung Adenocarcinoma	Improves apoptosis	[164]
*Salmonella* Typhimurium	*msbB*, *purI*	IDO ShRNA	Melanoma	Increase in ROS and cell death	[165]
*Salmonella* Typhimurium	*msbB*, *purI*	Sox2 shRNA	Lung Adenocarcinoma	I Inhibition of angiogenesis, increase in apoptosis	[166]
*Salmonella* Typhimurium	*hisG46*, *DEL407 aroA544::Tn10 (Tc^s^)*	MDR1 siRNA	Tongue Squamous cell carcinoma	Suppression of tumor proliferation	[167]
*Salmonella* Typhimurium	*hisG46*, *DEL407 aroA544::Tn10 (Tc^s^)*	MDR1 siRNA	Ovary Cancer	Slow tumor growth and sensitization to cisplatin	[168]
*Salmonella* Typhimurium	Chemical attenuation, UDP-glucose-4 epimerase	Survivan siRNA, GRIM-19	Larynx Cancer	Increase in apoptosis, inhibition of tumor growth	[169]
*Salmonella* Typhimurium	*aroA*, *LT2 Trp Met Erpsl* *flaA R-* *M+*	Bcl-2 shRNA	Melanoma	Delays tumor growth and prolongs survival	[170]
*Salmonella* Typhimurium	*phoP*, *phoQ*	Stat-3 shRNA	Larynx Cancer	Suppression of tumor growth	[171]
*Salmonella* Typhimurium	*PhoP*, *PhoQ*	Stat-3 SiRNA	Prostate cancer	Inhibition of tumor growth, decrease in metastases	[39]
**Nanomolecules**					
*Salmonella* Typhimurium	*purA::Tn10*	Liposomes loaded with doxorubicin	Triple negative murine breast cancer	Induces tumor cell death	[172]
*Salmonella* Typhimurium	*msbB*, *purI*	PLGA	Murine breast cancer	Improves the therapeutic efficiency of chemotherapy drugs	[173]
*Salmonella* Typhimurium	*msbB*	Liposomes loaded with doxorubicin	Murine colon cancer	Improves the selectivity and release of Dorubicin	[174]
*Salmonella* Typhimurium	*asd*	Nanoparticles loaded with indocyanine green	Murine melanoma	Inhibition of tumor growth	[175]
*Salmonella* Typhi	Chemical attenuation, UDP-glucose-4 epimerase	Gold nanoparticles covered in folic acid	Murine colon cancer	Enhances delivery of gold nanoparticles to the tumor	[176]
**Combination of *Salmonella enterica* and conventional antitumor treatments**					
*Salmonella* Typhimurium	*relA::cat*,*spoT::kan*	Radiotherapy	Colon cancer	Inhibition of tumor growth	[177]
*Salmonella* Typhimurium	*aroC*	CHOP	NHL	Increase in infiltrating lymphocytes, expression of cytokines and chemokines in tumor	[178]
*Salmonella* Typhimurium	*Leu*,*Arg*	Trastuzumab	Cervical cancer	Decrease in tumor volume	[179]
*Salmonella* Typhimurium	*Leu*,*Arg*	Chemotherapy (5-FU, cisplatin, gentamicin)	Pancreatic Cancer	Decrease in tumor	[180]
*Salmonella* Typhimurium	*Leu*,*Arg*	Cisplastin	Melanoma	Suppression of tumor growth	[79]
*Salmonella* Typhimurium	*Leu*,*Arg*	Recombinant methioninase	Metastasic osteosarcoma	Inhibition of tumor growth	[72]
*Salmonella* Typhimurium	Chemical attenuation, UDP-glucose-4 epimerase	Cisplatin	Prostate cancer	Induction of apoptosis	[181]
*Salmonella* Typhimurium	*purI*,*msbB* and *PhoP/PhoQ*	Chemotherapy (paclitaxel and doxorubicin)	Melanoma	Delays tumor growth and improves survival	[182]
*Salmonella* Typhimurium	*Leu*,*Arg*	Adaptive T-cell therapy	Fibrosarcoma	Tumor regression	[183]
*Salmonella* Typhimurium	*Leu*,*Arg*	Caffeine and valproic acid	Pleomorphic rhabdomyosarcoma	Inhibition of tumor growth	[184]

**Abbreviations:** PSA: Prostate-Specific Antigen; VEGFR-2: Vascular endothelial growth factor receptor 2; C-Raf: Serine-threonine kinases of the Raf family; NY-ESO1: New York Esophageal Squamous Cell Carcinoma-1; E7 (HPV16E7): Human papillomavirus protein E7; Melan-A: Melanoma Antigen; SVN: Survivin; HlyE: Haemolysin E; ClyA: Cytolysin A; PNP: Purine nucleoside phosphorylase; HSV-TK: Herpes simplex virus thymidine kinase; CPG2: carboxypeptidase G2; TGFα: Transforming growth factor alpha; PE38: Pseudomonas exotoxin A; CCL21: Chemokine (C-C motif) ligand 21; IL-2: Interleukin-2; IL-4: Interleukin-4; IL-18: Interleukin-18; IL-24: Interleukin-24; TRAIL: Tumor Necrosis Factor-Related Apoptosis-Inducing Ligand; VP3: apoptin; IFN-α: Interferon alpha; LIGHT: a member of TNF cytokine family; TNF-α: Tumour Necrosis Factor alpha; Laz: Lipidated azurin; FasL: Fas ligand; FADD: Fas-associated protein with death domain; Flt3: Flt3 Ligand; IL-15: Interleukin-15; Hsp70: Heat shock protein 70; TAA: Tumor-associated antigens; RBM5: RNA-binding motif protein 5; RNA: Ribonucleic acid; ShRNA: Short hairpin RNA; IDO: indoleamine 2,3-dioxygenase 1; Sox2: Sex determining Region Y-box 2; siRNA: small interference RNA; *MDR1*: Multipledrug resistance protein 1 gene; GRIM-19: Gene associated with retinoid-IFN-induced mortality 19; *Bcl-2*: B-cell lymphoma 2 gene; *Stat-3*: Signal transducer and activators of Transcription 3 gene; PLGA: Poly(lactic-co-glycolic acid); CHOP: cyclophosphamide, doxorubicin, vincristine and prednisone; 5-FU: 5-fluorouracil.

**Table 3:** Clinical trials using *Salmonella enterica* as cancer treatment.

Species	Mutation	Heterologous Molecule	Treated Malignancy	Dose and Administration	References
*Salmonella* Typhimurium	*Purl*, *msbB*	None	Phase I; Metastatic melanoma, metastatic renal carcinoma	Intravenous 1 × 10^6^, 1 × 10^9^ CFU, single escalating dosing	[29]
*Salmonella* Typhimurium	*Purl*, *msbB*	IL-2	Phase I, Metastatic liver carcinoma	Oral, escalating dosing with 1 × 10^5^, 1 × 10^10^ CFU per dose	[214]
*Salmonella* Typhimurium	*Purl*, *msbB*	Cytosine deaminase	Phase I; Head and neck carcinoma/esophageal adenocarcinoma	Intratumoral injection of 3 × 10^6^, 1 × 10^7^, 3 × 10^7^ CFU/m^2^ at escalating dosing, for various cycles	[208]
*Salmonella* Typhimurium	*Purl*, *msbB*	None	Phase I; superficial solid tumors	Intratumoral injection of 3 escalating doses	[215]
*Salmonella* Typhimurium	*Purl*, *msbB*	None	Phase I; metastatic cancers	Intravenously with escalating doses every 35 days	[216]
*Salmonella* Typhimurium	*Purl*, *msbB*	None	Phase I, non-specific solid tumors	Intravenously with escalating dosing every 35 days	[217]
*Salmonella* Typhi	*galE*, *rpoS*, *ilvD*	VEGFR-2	Pancreatic cancer	10^6^ to 10^10^ CFU Single dose	[126]
*Salmonella* Typhi	*galE*, *rpoS*, *ilvD*	None	Phase I: non-muscular bladder carcinoma		[218]
*Salmonella* Typhimurium	*---*	Neuroblastoma-associated antigen and protein of the potato virus X	Pilot study Recruitment period Neuroblastoma	10^10^ CFU orally, at 1-week intervals, for 3–4 weeks	[219]
*Salmonella* Typhi	*---*	Survivin	Pilot study Multiple myeloma	2 escalating doses every 2 weeks 2.5 × 10^6^–2.5 × 10^7^	[220]
*Salmonella* Typhimurium	*Asd*, *cAMP y* receptor de cAMP	IL-2	Metastatic pancreatic cancer	2.5 × 10^6^ CFU every week for 6 weeks, orally	[212]
*Salmonella* Typhimurium	*Purl*, *msbB*	L-methioninase	Refractory solid tumors	0.9–2.0 × 10^9^ CFU, intravenously	[209]
*Salmonella* Typhimurium	*Purl*, *msbB*	Cytosine deaminase	Metastatic cancer	2.5 × 10^6^ CFU in mice. 1 × 10^10^ CFU in primates. Intravenous or intratumoral	[192]
*Salmonella* Typhimurium	*Purl*, *msbB*	L-methioninase	Head and neck advanced squamous cell carcinoma	0.9–2.0 × 10^6^ CFU Intratumoral	[210]
*Salmonella* Typhi	*galE*, *rpoS*, *ilvD*	VEGF	Pancreatic cancer	10^6^ or 10^7^ CFU, orally	[211]
*Salmonella* Typhimurium	*Asd*, *cAMP* and *cAMP receptor*	IL-2	Liver metastatic solid tumor	10^10^ CFU, Orally	[213]

Abbreviations: IL-2: Interleukin-2; VEGFR-2: Vascular endothelial growth factor receptor 2; CFU: Colony-forming unit.
